# Medical Topography of Florida

**Published:** 1861-07

**Authors:** G. R. B. Horner

**Affiliations:** Surgeon U. S. N.


### ART. I.—*Medical Topography of Florida.* By G. R. B. HORNER, Surgeon U. S. N.

THIS State, though seceded from the Union, is still worthy of attention. Situated between 25° and 31° north latitude and 81° and 87° west longitude, having the Atlantic on the east side and the Gulf of Mexico on the south, she enjoys an equable and temperate climate. Her shores are fanned daily by sea breezes, and at night the land breeze usually prevails. Copious showers fall in autumn and winter, and commonly, also, in July and August. The most frequent and heavy are in winter, but constant rain and days uniformly cloudy rarely occur.

The following may be set down as the ordinary condition of the air as indicated in West Florida:—

| | | | |
|---|---|---|---|
| Barometer—Average | | 30 | inches. |
| | Maximum | 30·56 | " |
| | Minimum | 29·65 | " |
| Thermometer—Average | | 60 | degrees. |
| | Maximum | 96 | " |
| | Minimum | 19 | " |

But I have heard of the thermometer being lower. No snow fell, to my knowledge, during my residence of more than ten years at the Naval Hospital near Warrington; though during the winter of 1859–60, the weather was as cold as it had been for many years, and ice an inch thick formed.

The summer lasts from seven to nine months, and near the Gulf is very sultry, the air being highly charged with moisture, and mould and rust forming on all things liable to them. So great is the dampness of the air, sometimes even in winter, that plank is substituted for plaster in public and private residences, and walls not lathed and plastered precipitate moisture or steam to such an extent as to form streams trickling down them to the floors. From a mantle-piece, two years ago, I twice sponged a half-pint of water, which had come down from the unlathed face of a brick chimney in my chamber. This occurred during mild after cold weather, which had chilled its walls. In East Florida the northeast trade-wind blows constantly on the Atlantic coast, especially at the southern end of the peninsula and the reefs and islands running to the southward and westward. Hence, at Key West, this wind is used for pumping sea water into high wooden pans, by means of windmills, for the manufacture of salt. The total of this made at the pans of Colonel Dennis, at the eastern end of the island, had reached 60,000 bushels per annum, when I was there three years ago, inclusive of the salt obtained by simply letting the sea water evaporate in pans filled by the tides. At Warrington and Pensacola the sea breeze regularly arises in the south during the morning, blows afterward strongly from southwest, and, dying away in the evening, hauls around to the west. During the night the land breeze springs up in the northwest and blows until morning, rendering the night cool—sometimes uncomfortably so—and it is thought unhealthy by many persons, from that wind blowing across the marshes and low grounds of the interior of the country. Most, if not all, of the western part of this region may be correctly described as low, undulating, and sandy, covered uniformly with dense forests of yellow pine, interspersed with dwarf black oaks and open marshy spaces, termed savannas, often filled with water in winter. But these oaks scarcely appear above the ground until the pines are cut or burnt down, and they then spring up, grow thickly, and attain a height of ten or fifteen feet on an average. Near the sea the live oak is also found growing either among the pines or together, and attain vast size after many years' growth. In a yard, near Fort Barrancas, may be seen some about ten feet around the trunk, with huge craggy branches spreading out their small oval leaves for 150 or 200 feet around. The yellow pine is converted into plank and timbers, and forms the most important article of commerce in West Florida. The live oak was carefully nursed by our government, for the use of the navy, until secession occurred, and several persons were paid salaries as inspectors or superintendents of its live oak lands. Along the western shores of the Gulf the land consists of white sand without shells or pebbles; not a stone is to be seen on them; and the only ones observed by me were some sandstones in the pine forest on the road from Mobile to Pensacola. But Key West and other islands adjacent, those at Cedar Key at the junction of the peninsula of East with West Florida, are formed of shells, crude or broken, or in powder like fine white sand, and of coral. So great is the quantity of these calcareous substances that the shoals at Key West seem to be of chalk and water mingled. In these shoals sponges, green-turtle, king-fish, a large eel of fine flavor, conchs, and many other soft and shell fishes abound.

As the sea-coast of East Florida differs from that of West Florida, so does the interior; for in the former limestone of secondary formation is plentiful. Vast quantities are seen under and at the sides of the railroad between Cedar Key and Fernandina, distant 155 miles; and the swampy lands are very rich. In their natural state they form dense forests of cypress, oak, sweet gum, magnolia, cabbage-tree, maple, and other trees; they have, besides, an undergrowth of palmetto grass, whose rough roots, of size sufficient for large trees, render the clearing of the ground for cultivation very difficult and expensive. But the enterprising farmers from South Carolina and other States have succeeded in making many plantations for the cultivation of cotton, sugar, and corn, and East Florida has become a formidable rival to other Southern States in those products.

As I passed over the above road, I was told of one plantation south of it which had four hundred slaves employed on it; and the best cotton I have seen in the South was upon this western side of the road, near its junction with the railroad to Jacksonville.

Agreeably to the statement of the late talented surgeon, Bernard M. Byrne, of our army, who died at Fort Moultrie last September, of yellow fever, "three hogsheads of sugar have been made on one acre of high hummock land, after it had been six years in successive crops of corn, without the aid of manure." These swamps form the most durably rich lands, and next to them are what are termed hummocks, which are low or undulating, high, dry, and covered with luxuriant oaks and other trees. The pine land of Florida is divided into three kinds, the richest of which, he affirms, has nothing analogous to it in any of the other States, its surface being covered for several inches in depth with a dark vegetable mould, beneath which, for several feet, is a chocolate-colored sandy loam mixed, for the most part, with limestone pebbles, and resting on a substratum of marl-clay, or limestone-rock.

Besides the finest trees mentioned, the cedar, odoriferous bay, pride of China, the titis, remarkable for its small leaves and still smaller beautiful flowers, the water-oak, and various other trees, are found in West Florida. Among them are the persimmon, French fig, and orange, which grows kindly and produces abundantly, though occasionally killed by intense cold. Its fruit has also been very much injured by a very small black insect infesting its cuticle. The wild plum is produced abundantly, and when cultivated is excellent. The fruit of the persimmon, when ripe, is sweet, luscious, and not excelled by the oriental date. Both of these fruit-trees grow spontaneously. Chinquepins, blackberries, gallberries, the stramonium, pokeberries, and wormseed are products of the soil, and the plants of the two latter grow most luxuriantly and spontaneously wherever the seed are scattered. In spring the poke forms one of the best vegetables to be had in the country. By cultivation and frequent irrigation peas, beans, cabbages, turnips, radishes, Indian-corn, arrow-root, cantalopes, pumpkins, and water-melons are obtained, as are likewise potatoes and tomatoes. Indeed, there is no doubt that when Florida becomes thickly populated its productions will be vastly increased, and exceeded by few countries in the world; for there is no season when certain fruits and vegetables may not be raised on her soil. She has, moreover, a great advantage over the Western States by having her fine forests undergrown with grass, which affords pasturage for horses, sheep, deer, and cattle throughout the year. It grows spontaneously, and is rendered more abundant by being burnt when dry. This is, therefore, yearly done by graziers, though the forests are often consumed or killed by the flames of the burning grass. The numerous ponds of water, the frequent rains, and copious streams of water from innumerable springs afford still greater facilities for grazing. The rareness of tornadoes, violent and long-continued storms, and long droughts render Florida more desirable to agriculturists than Texas, and other portions of America subject to them.

In mineral wealth Florida is very deficient, but her animal kingdom is decidedly wealthy in some respects. Her ponds, lakes, rivers, and bays abound in fish and birds. Among the former are the trout, mullet, red and black snappers, the favorite pompeno, sheepshead and Spanish mackerel, catfish, flounders, perch, and a variety of shellfish, of which the oyster is most abundant. The porpoise, shark, and devilfish, the largest of the flat ones, should also be enumerated. The common fresh water turtle, the green sea turtle, and crabs abound. In the sandy ridges and pine forests and away from water are found burrowing the land turtle or terrapin, called gopher—a very poor and ugly one—but when first roasted or fried and boiled, forms an excellent soup, much used during warm weather, when the gopher is most abundant.

*Birds.*—Besides the ordinary domestic ones are many which are always found, and others to be met with during winter, when the cold in the Northern States forces them South. The chief birds I saw were doves, crows, hawks, owls, gulls, snipes, partridges, sparrows, robins, woodpeckers, buzzards, mocking, blue, and jay birds. Eagles, thrush, wild ducks, cranes, swallows, martins, whip-poor-wills, and paroquets are likewise found; but the only ones of the last named I saw were in East Florida.

Hares, oppossums, raccoons, squirrels, and deer are the chief quadrupeds hunted. Bears, wolves, foxes, and moles are also found.

Insects and reptiles are abundant. Lizards, alligators, black adders, vipers, moccasins, and chasers; the small ground rattlesnake, which has no real rattles, and the large rattlesnakes are very numerous. The latter attain an enormous size, being sometimes six or nine feet long, and of corresponding thickness. So numerous are these snakes that even in winter persons must be cautious in going into places where they may be concealed, or come forth to bask in sunshine. They feed on young hares, rats, and frogs which abound in swamps or branches, and when night comes on croak in concert; while the locust-like green insects, called katydids, sing in the adjacent trees. Among frogs the most remarkable are those living on trees, and having feet with suckers, by which they climb and hold on with great ease. After showers they appear in great numbers, and make the air resound with their singular notes. Beetles, ants of many kinds, including the large blood-red ones, spiders, sand flies, the small yellow-horned ones, and musquitoes are countless, and it is hard to determine which of the two last named bite the most fiercely. The common house flies are scarce.

*Diseases.*—From my observations, as well as those of others, malarious fevers may be correctly asserted to be the most common affections in Florida, near the marshes and fresh water ponds and streams; but away from such localities they are rare, and the inhabitants generally enjoy as great exemption from those diseases and others, as perhaps any people in our country. Yellow fever occurs from time to time, but is said to have been always from importation, particularly from New Orleans, Mobile, and the West Indies. At Warrington, it is stated, it has happened regularly, at intervals of seven years, for a considerable period; but this did not prove true last year, (1860,) when no cases of yellow fever occurred, though it was the seventh year since the great epidemic of 1853, when this fever was so fatal there, and created a general belief that it was contagious, as there was the strongest evidence of its having been communicated to sailors, soldiers, nurses, and civilians in the navy-yard, town, naval hospital, and military barracks at Fort Barrancas, by their association and being confined in the same apartments. On this subject I made special inquiries of the citizens and Dr. Bishop of the navy, as well as of Dr. Hargis, of Pensacola, and they fully confirmed the statements of Assistant Surgeon John F. Hammond, in his report to Surgeon-General Lawson, in 1854, regarding the prevalence, causes, and treatment of this fever, and other medical subjects. Dr. Hammond states that "the apparent contagiousness of the disease was a subject of general remark, and not a question." But he remarks afterward: "It may be that during the epidemic of this season (1853) there was a mild form of typhus fever, running its course at the same time with yellow fever, tending to contagiousness." Dr. Hargis, of Pensacola, related verbally to me a number of facts to prove that yellow fever was contagious there in the above year, and told me of a mattress, on which a case had ended fatally, near Milton, in September, having been sold in December following, and communicated the disease to the person who subsequently slept upon it. These and similar facts the doctor published in the *New Orleans Medical and Surgical Journal.* The late Dr. George Tyrrel, surgeon to the Warrington Naval Hospital in 1853, likewise reported, on the 4th of September, to Surgeon Thomas Harris, chief of the bureau of medicine and surgery, concerning yellow fever, that "it is evidently epidemic ; but we have strong reasons to believe it is also contagious. The nurses have most of them had it, and several of them are now down. Invalids who have been employed in attendance upon the sick, have been attacked, and in one instance two patients, occupying beds on each side of a man who died of the disease, were seized with it. The steward of the hospital has been very ill, and Dr. Jeffrey and myself have been slightly indisposed." Finally, Surgeon Byrne states, in his report of November 10th, 1858, to Surgeon-General Lawson, relating to the introduction and prevalence of yellow fever among the troops at Fort Moultrie : "It seems to me that if we regard yellow fever as a contagious disease—a fact of which I entertain no doubt, and the truth of which I shall endeavor to prove on another occasion—the facts here stated will satisfactorily account for its spread on a sandy, dry, healthy island, where no local cause of disease could be discovered, but where the population was much larger than it had been before, and the residences more crowded than they usually are in the largest cities."

During my stay in Florida no instances of this fever were under my care. One or more were reported to be in or about Warrington; but one was said to have been that of a man from Mobile; and of sixty cases of remittent and intermittent treated in the hospital while in my charge, a majority were brought to it from ships of war arrived from cruising on the West India station, including Mexico and the coast of Central America. The remaining cases were sent from the navy-yard, and belonged to it and the marine barracks.

*Treatment.*—This consisted principally, in these cases, of acidulated and hot drinks, according as the fever was in the hot or cold stage ; of the neutral mixture, made with bicarbonate of potassa or carbonate of ammonia, with from one to two grains of tartar emetic in it ; of blue pill and calomel, when the tongue was furred or the liver affected ; and of the sulphate of quinine, chiefly in solution, sometimes in pills, and in from one to five grain doses, during remissions and intermissions. When the stomach was irritable, a blister was put over it, and, the cuticle having been raised, the quinine was finely powdered and sprinkled upon the raw surface beneath. For severe palpitations of the heart, which were frequent, the tincture of digitalis and tincture of veratria viride were given; the latter in from three to five drops in solution, and used effectually.

Of the cases of remittent fever six came from the United States ship Fulton; and four of them, having a typhoid character, were treated correspondingly. Her commander, Captain G. G. Williamson, died of this fever at the navy-yard, having vomited incessantly. Indeed, it was believed that had not the vessel been wrecked on the coast of Santa Rosa Island, and her coal been taken out of her, the fever would have become decidedly typhus icterodes.

Next to miasmatic fevers, diarrhœa, dysentery, catarrhs, pulmonic diseases, rheumatism, and eruptions prevailed, especially during the summer and winter solstice. Among the children of Warrington and Walsey, a village east of the navy-yard, cholera and convulsions were very common, and frequently fatal. Dysentery among adults is often destructive, but the too free use of calomel in its treatment, and other improper remedies, may rationally be assigned as a principal cause of its mortality. Hiccough is one of the most ominous symptoms. In the treatment of this affection, anodyne enemata, and tannin with opium, followed with aniseseed oil, were the most effectual remedies. Sometimes pills of ipecacuanha, opium, and calomel were given, and in all cases the patients were subsisted chiefly on well-boiled rice-water, arrow-root, and soft-boiled eggs, during convalescence. In a very bad case, accompanied with hiccough, which had resisted every other means, this fatal symptom was relieved by the inhalation of chloroform, and the patient entirely recovered soon afterward.

Catarrhs, bronchitis, and other pulmonic disorders prevail during the chilly, damp weather of winter, and rheumatism is common at the same time along the coast of West Florida. Hence invalids, who either live on it permanently or have resorted there to be relieved of the above affections, are liable to have them increased. During the sultry weather of summer, cutaneous diseases prevail, especially among strangers, and even among old residents of sanguine temperaments, much exposed to the sun. Boils, carbuncles, and lichen tropicus are the most common. Carbuncles are sometimes very severe and fatal. Soon after my arrival at the hospital I was called in consultation to a merchant who had one the size of a hen's egg over the left eye, another which had partly destroyed the palm of one hand, and a third one so large as to occupy the back and one side of his neck. He was a large man, of sanguine temperament and intemperate habits, and was also suffering from lichen tropicus. So malignant were the carbuncles that no remedies restrained them. Blue mass, morphia, quinia, and guaiac internally, with bitter infusion, and linseed poultices, solutions of acetate of lead, sulphate of zinc and caustic potassa, frequent lancing, and other means, were used in vain. The fingers of his wife became sore from dressing the carbuncles, and he at last died. It may be well, moreover, to state that, having myself been previously affected with lichen tropicus on the coast of Central America, and likewise in Florida from the period of my arrival, I finally cured the disease by the use of aqua ammonia, except between my shoulders; an enormous carbuncle appeared there a few days after my patient's death, and was not cured until the lapse of nearly two months. Besides the above lotions, I found a solution of nitric acid, in the proportion of one drachm to two ounces of water, one of the most efficacious to remove fungus and heal up the parts. Blue mass, and solution of sulphate of quinia in elixir of vitriol and water, to promote the secretion of bile, and restore strength, were frequently employed. At first, there having been loss of appetite and slight fever, a digestible diet, chicken broth, bread, and coffee, constituted my chief sustenance. In the treatment of carbuncles I found that after a certain time, that is, when suppuration had been established, warm poultices rather caused an extension of the disease, and it was best to leave them off and restrain inflammation adjacent, with frequent applications of lead-water and a dressing of simple cerate, mingled with a few grains in each ounce, and spread upon a bit of old muslin.

*Naval Hospital.*—This spacious, airy, and elegant structure, founded in 1827, and finished in 1846, is situated eight miles from Pensacola, close to Warrington—which lies between the hospital and the navy-yard—and within sight of Forts Pickens, McRea, and Barrancas. From the two former the hospital is separated by the lower part of Pensacola Bay, and from Fort Barrancas and its barracks by a forest of live oaks and pines which extends backward of them for many miles. The hospital stands upon a bluff of yellow sand thirty feet high, some hundred yards back from the beach, and westward a half-mile to the latter fort. All the land occupied by the above-named works, as well as the intervening country as far back as the great bayou between the navy-yard and Pensacola, are reserved territory of the United States, and were never ceded to Florida, although now in her possession and that of the other cotton States.

The hospital and buildings attached to it occupy twelve acres of ground, inclosed within a substantial brick wall eleven feet high, resting on a stone foundation. During my term of service there I had fourteen acres more inclosed in two lots—one of about four, the other of ten acres—between the front wall and back one, and having Preston Avenue, or the main street of Warrington, passing between the two lots. These were cleared of swamps, leveled, adorned with additional trees, forming an avenue to the water, and planted with the Bermuda grass, which grows by joints, sends down a root from every one, has great vitality, and is an admirable grass for a sandy soil. Hence, it is invaluable in forming banks and terraces. To cause it to grow it is simply dug up and buried at intervals of a few feet in the sand; if the season be favorable the young blades soon spring up, the roots shoot forth from every joint and penetrate to a great depth, while their horizontal stems form a perfect net-work. If this grass come in contact with a fence—wooden or stone—it spreads underneath, and sometimes grows upward, like a vine, for several yards. So deep does it grow, and such is its tenacity of life, that, once well rooted, even in the poorest white sand, it is hardly possible to eradicate it permanently.

The hospital, situated southwest from Fort Pickens, consists of a main building and two wings, presenting a front of 400 feet. The main edifice is three stories high, surmounted by a large cupola resting on pillars, and commanding a prospect of the Gulf of Mexico, forts, and harbor; the towns of Walsey and Warrington, with the navy-yard between them; a distant view of Pensacola and the country back of the harbor and sea, and the Island of Santa Rosa, stretching for forty miles to the eastward. The wings are two stories high, and each story, as well as the main building, has a portico thirteen feet wide on every side. The pillars of the first story are square, and made of brick; those of the upper are of wood, handsomely turned, and sixteen feet high. The doors and windows open into the porticoes and extend to the floor, so that, when open, the ventilation is perfect. The main building has the kitchen; two dining-rooms and a bath are in the first story; a spacious parlor, and dining-room for officers, a dispensary and library in the second story, and two fine wards in the third one. In the garret are four large tanks lined with zinc, and filled with water, by a steam-engine, from an artificial pond in front of the west wing. The pond is supplied from a spring at the foot of the bluff and west of the hospital, as well as from the drainage from the former. The tanks furnish water for the kitchen, baths, and dispensary. Drinking water is chiefly supplied by rain collected on the slate roof, and conducted by pipes into two large cisterns, one in the first story of the main building, the other in that of the eastern wing. This and the western contain the smaller wards, cells, rooms for officers who are sick, and attendants, and a small museum I was forming, when detached, and containing a collection of Floridian curiosities. The whole hospital is heated by fuel burnt in open fire-places. The library was formed about fifteen years ago, chiefly by contributions in books and money from men and officers belonging to the Warrington station, from citizens in various parts of the Union, and from members of Congress, and our government. The two largest contributors in money were the late Surgeon Francis Hulse and George Tyrrel; but to the former is principally due the establishment of the library. Hence it has been given his name. Its contents consist of upwards of 1400 volumes, among which are many of ancient and modern history, the Family Library, Sir Walter Scott's novels, many other interesting works, and some standard medical and surgical treatises. The library having been formed mainly for the amusement of invalids, the latter works were very deficient when I took charge of the hospital, and while there, I appropriated nearly all the income of the library in supplying the deficiency.

All the wards and chambers are furnished with iron bedsteads, two hundred and fifty in number—including some in the pest hospital—but many more could be accommodated. Each bedstead is supplied with a hair mattress and musquito bar, which is absolutely necessary during warm weather. The latter hospital, built about twenty years since, and often termed French from being first occupied by invalids from the French squadron in the Gulf of Mexico, stands back of the western wing. It is made of the same materials, and after a similar style to the chief hospital, having porticoes all around each of its two stories. The first one contains the cells, rooms for officers and attendants; the second story contains two pairs of handsome wards communicating by folding-doors; water was supplied from a brick tank in one corner, but it having destroyed the adjacent floor, it was converted by me into a store-room, and a well back of this hospital now supplies its water. Having none, I had an ordinary large brick privy built for it; but the chief hospital has two of much greater size, two stories high, with tanks in their garrets, and standing back of each wing. An arched brick culvert acts as a drain to these privies, and carries its refuse into the forest east of the hospital. For the interment of the deceased there is a cemetery of three acres of land inclosed by a new brick wall, and situated some hundred yards north of the hospital grounds. Within these, at the back, stand an ample, handsome stable adorned with a portico in its whole length, and other out-houses, and just within the hospital walls, eastward and westward, are the dwellings of the surgeon and assistants, built in the same fine style as the former and separated from its yard by picket fences, inclosing from two to three acres for each building, with groves of live oaks, some pines, and other trees, such as adorn the hospital yard and the lots, intervening between it and the harbor. Beneath the shade of these trees were formerly seen walking or resting the sick and wounded, luxuriating in the refreshing breezes which, every day in summer, blow from the Gulf from morning until evening. Now, our seamen, when sick, can no longer resort to this hospital, as it is occupied by the invading forces of the Southern Confederacy.

